# The Effect of Moderate-Intensity Treadmill Exercise on Bone Mass and the Transcription of Peripheral Blood Mononuclear Cells in Ovariectomized Rats

**DOI:** 10.3389/fphys.2021.729910

**Published:** 2021-10-29

**Authors:** Li Gao, Ye Li, Yong-Jie Yang, Dong-Yan Zhang

**Affiliations:** College of Sports and Health, Shandong Sport University, Jinan, China

**Keywords:** ovariectomized rats, moderate intensity treadmill exercise, bone mineral density, bone microstructure, peripheral blood mononuclear cells, transcriptome sequencing

## Abstract

**Objective:** Using RNA-sequencing technology to screen the effect of moderate-intensity treadmill exercise on the sensitive genes that affect bone mass in the peripheral blood mononuclear cells (PBMCs) of ovariectomized (OVX) rats.

**Methods:** Three-month-old female Sprague-Dawley rats of Specific Pathogen Free (SPF) grade were randomly divided into the sham operation (SHAM) group, OVX group, and OVX combined exercise (OVX + EX) group. The OVX + EX group performed moderate-intensity treadmill exercise for 17 weeks. Then, the body composition and bone mineral density (BMD) were measured, and the bone microstructure of the femur was observed. PBMCs were collected from the abdominal aorta, and the differential genes were analyzed by transcriptome sequencing to further screen sensitive genes.

**Results:** (1) In the OVX group, the body weight and body fat content were significantly higher than in the SHAM group while the muscle content and BMD were significantly lower than the SHAM group. (2) The trabecular bone parameters in the OVX group were significantly lower than in the SHAM group, and they were significantly higher in the OVX + EX group than in the OVX group. When compared with the SHAM group, the microstructure of the distal femur trabecular in the OVX group was severely damaged, suggest that the morphological structure of trabecular bone is severely damaged, the number of trabecular bones is reduced, and the thickness becomes thinner, which lead to the widening of the trabecular bone space and the appearance of osteoporosis. The number and continuity of the trabecular bones were higher in the OVX + EX group than in the OVX group. (3) A Venn diagram showed that there were 58 common differential genes, and the differential genes were mainly enriched in the PI3K-Akt signaling pathway. Five sensitive genes were screened including CCL2, Nos3, Tgfb3, ITGb4, and LpL. The expression of CCL2, Nos3, and Tgfb3 genes was closely related to multiple bone parameters.

**Conclusion:** Moderate-intensity treadmill exercise may improve the body composition and bone mass of the OVX group by upregulating CCL2 and other genes of the PBMC. The PBMCs in the peripheral blood can be a useful tool for monitoring the effect of exercise on bone health in postmenopausal osteoporosis.

## Introduction

Postmenopausal osteoporosis (PMOP) is a common disease in middle-aged and elderly women, with a high incidence, significant harm, and difficult treatment. Its prevention and treatment have become important topics of global concern. A reasonable and appropriate amount of physical exercise has been proven over many years of clinical practice to have great benefits for the prevention or treatment of PMOP (Tartibian et al., 2011; Harding and Beck, 2017; Cunha et al., 2018). However, bone density does not fully reflect bone quality (Hernandez and Keaveny, 2006; McNamara, 2010). Mechanical load can affect bone strength by changing the geometry of bones, inducing osteoblasts to express cytokines, and inhibiting osteoclast differentiation and bone activity and absorption, thereby affecting the bone remodeling process, but the specific mechanism of action is still unclear. Arron and Choi (2000) first proposed that bone immunology plays an important role in bone metabolism and believed that bone is an immune organ. A variety of immune cells originate in the bone marrow, and bone tissue cells share the same bone marrow microenvironment, regulatory factors, and receptors. Subsequently, Srivastava et al. (2018) and Tilkeridis et al. (2019) proposed the concept of “immunoporosis,” further revealing the regulatory mechanism of immunology in osteoporosis. Bone immunology revealed that the immune system, including T cells, B cells, and inflammatory cytokines, is a key regulator of osteoclasts and osteoblasts. Increasing evidence shows that the immune system and the skeletal system not only share cytokines but also a variety of signal molecules, transcription factors, and membrane receptors (Liu H. et al., 2017). Peripheral blood mononuclear cells (PBMCs) mainly refer to lymphocytes and monocytes and are the only source of osteoclasts in adults. PBMCs first form immature multinucleated osteoclasts, which then migrate to the site where bone resorption occurs and are activated and transformed into mature osteoclasts (Zhou et al., 2015). In addition, activated T cells and macrophages can affect the activity of osteoclasts and their precursor cells (Takayanagi et al., 2000). There are molecular markers for osteoblast precursors that account for 1–2% of monocytes in the blood, and their number in adolescent boys is five times that of adult individuals (Eghbali-Fatourechi et al., 2005). Li Y. et al. (2007) showed that lymphocytes are the key stabilizers of basic bone turnover and the key regulators of peak bone mass in the body. T lymphocytes, B lymphocytes, cytokines, chemokines, and costimulatory molecules can all interact with osteoblasts and osteoclasts to jointly regulate bone formation and bone resorption, thereby regulating the bone remodeling process. Due to the limitation of bone mineralization and human bone tissue extraction, it is difficult to directly study the effect of exercise on the function of human bone cells. PBMCs are closely related to bone changes, extraction and purification methods are relatively mature, and in recent years, PBMCs have gradually become widely accepted for use in investigating the biological mechanism of PMOP (Daswani and Ikram Khatkhatay, 2018; Zhang et al., 2020). RNA sequencing (RNA-seq) is based on the Illumina HiSeq high-throughput sequencing platform to sequence and analyze all messenger RNA (mRNA) sets transcribed by specific tissues or cells at a certain time and can comprehensively and rapidly obtain an abundance of information about the mRNA. RNA-seq is currently an important method for exploring the molecular mechanisms of diseases. In this study, three-month-old ovariectomized (OVX) rats were used to simulate a PMOP model, and a 17-week moderate-intensity treadmill exercise intervention was applied. An RNA-seq bioinformatics analysis of the PBMCs was carried out to screen for genes sensitive to exercise that reflect bone changes. From the perspective of transcriptomics, the molecular mechanism of exercise in slowing down the bone loss caused by an estrogen deficiency in elderly women has been revealed, which provides a theoretical basis for exploring the molecular mechanism of exercise in delaying PMOP.

## Materials and Methods

The research design and procedures in this study were in line with the Helsinki Declaration, and the experimental procedure was approved by the Ethics Committee of the Shandong Institute of Physical Education (approval no. 2018208). During the experiment, every effort was made to minimize discomfort and pain, and sedation, analgesia, anesthesia, or pain-free execution were employed as needed.

### Study Objects and Groups

A total of 60 three-month-old female Sprague–Dawley rats of SPF grade were purchased from the Beijing Vital River Laboratory Animal Technology Co., Ltd., (license no. SCXK [Beijing] 2016-0006). The rats weighed 272 ± 14 g, were raised in a single cage under artificially controlled indoor lighting in a 12 h light and 12 h dark environment, were given free access to food and water, and were fed with the conventional national standard rodent feed. The ambient temperature was 22–26°C, and the relative humidity was 45–70%. The rats were randomly divided into the OVX (*n* = 40) and the sham operation (SHAM; *n* = 20) groups. Bilateral dorsal ovariectomies were performed in the OVX group (Park et al., 2010). One week after surgery, 20 rats from the OVX group were randomly selected and placed into the OVX combined exercise (OVX + EX) group.

### Model Preparation

All the rats were fasted for 10–12 h. All the experimental rats were anesthetized using an intraperitoneal injection of a general anesthetic agent (3% pentobarbital sodium [1 ml/kg]), laterally positioned, and disinfected with iodine and alcohol. A longitudinal incision was made 0.5–1.0 cm adjacent to the midline of the bilateral back of the rats. The skin, fascia, and muscle were separated to both sides, and the weakest parts of the left and right abdominal muscles were opened. The pink ovaries were visible below the kidneys on both sides, and the ovaries were gently lifted. After ligation, the ovaries were removed, and the skin was sutured. In the SHAM group, a sham operation was performed in which the hair was removed, an incision was made on the skin in the center of the back in the same manner as in the OVX group, and the site was sutured after removing the large, white adipose tissue adjacent to the ovaries.

### Motion Scheme

The rats were rested for 1 week postoperatively, iodine tincture was smeared on the wound daily, and penicillin injections were administered for three consecutive days to prevent infection. With reference to the standard load model adopted in the relevant literature (Liu H. et al., 2017), a load program of moderate-intensity treadmill exercise was formulated. In the second week after the operation, the rats in OVX + EX group received adaptive training at a fixed time every day, and in the third week after the operation, the rats were trained at 20 m/min for 60 min until the end of exercise program. All training occurred in the morning, 5 days per week for 17 weeks.

### Measuring Indexes

#### Determination of the Body Composition and Bone Mineral Density (BMD) of the Rats

**FIGURE 1:**
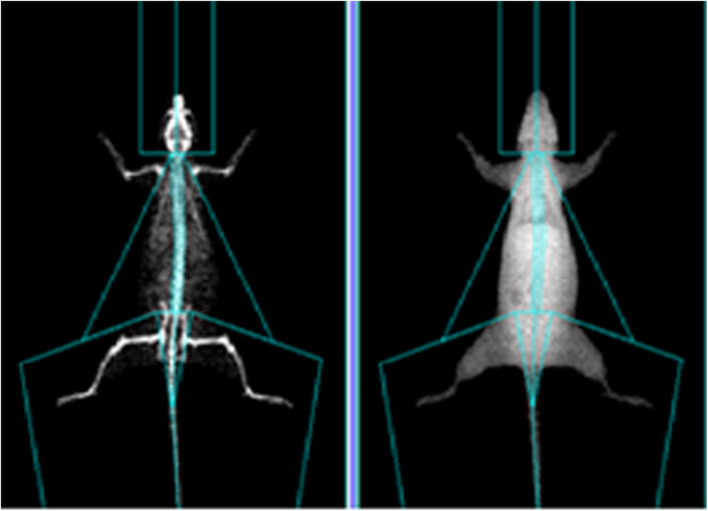
Representative images of DEXA analyses.

All the rats were weighed and anesthetized before sampling. Each rat was divided into parts based on the anatomical structure of the rats (see Figure 1) to calculate the percentage of upper limb, lower limb, trunk, and whole-body muscle and fat, which was calculated as muscle (or fat) mass ÷ body weight. After anesthesia, the rats were measured by selecting the small animal measurement model of the Prodigy dual-energy X-ray absorptiometry (Lunar product division, GE Healthcare, United States). In order to prevent the last exercise from interfering with the experimental results, the rats in the OVX + EX group were sacrificed 24–36 h after the last training. The abdominal cavity was then opened quickly to expose the abdominal aorta, and blood was collected for the extraction of the PBMCs. Subsequently, the rats were euthanized; the left femur, tibia, and fourth lumbar vertebra were separated; and the soft tissues were removed. The tissues were wrapped in gauze soaked with normal saline and placed in a test tube. After labeling, the tissues were frozen at −80°C for the determination of the BMD. The Norland XR-600 bone densitometer was used to measure the BMD and bone mineral content in the distal femur, proximal tibia, and fourth lumbar vertebra. The right femur was separated and fixed with 4% paraformaldehyde to observe the bone microstructure.

#### Micro-Computed Tomography (micro-CT) Scanning of the Femurs

Four right femurs were randomly selected from each group, and the distal femurs were scanned using the SkyScan 1172 Microtomography. The samples were placed along the long axis of the scanning bed to ensure that they were placed in the same direction and on the same horizontal plane, and scanning was started with a voltage of 80 kV, a current of 112 μA, and a pixel density of 9.00 × 9.00 μm. After scanning, the samples were reconstructed. The region of interest was located between the disappearance of the distal growth plate of the femur and 100 layers above it. The threshold value was automatically generated by the computer, the image information was extracted, and the image binarization was completed. Finally, a quantitative analysis was carried out using software to obtain the indicators for the microstructure of the distal femurs of the rats including percentage of bone volume (BV ÷ total volume [TV];%), mean total cross-sectional tissue area (μm^–2^), mean total cross-sectional tissue perimeter (μm), mean total cross-sectional bone area (μm^–2^), mean total cross-sectional bone perimeter (μm), mean number of objects per slice, closed porosity (%), trabecular thickness (plate model; Tb.Th; μm), trabecular separation (plate and rod model; Tb.Sp; μm), trabecular number (plate and rod model; Tb.N; μm^–1^), trabecular diameter (rod model; Tb.Dm; μm), and mean trabecular pattern factor (Tb.Pf; μm^–1^). At the same time, three-dimensional reconstruction images were created.

#### Collection, Extraction, and Transcriptome Analysis of the PBMCs

The blood of four rats (2.5 mL each) was mixed in equal amounts. The peripheral blood PBMCs were extracted using the rat mononuclear cell extract (Tianjin Haoyang Commercial Co., Ltd.), 0.5 mL of Trizol was added, and the result was stored at −80°C. The total RNA was extracted, and after the purity, concentration, and integrity of the sample were qualified, the library was constructed. Based on the Illumina sequencing platform, the double-end (paired-end) sequencing method was used to complete the transcriptome sequencing analysis of the PBMCs of the rats. The differentially expressed genes were screened with a fold change (FC) > 2 and *P* < 0.05. The Venny 2.1 website^1^ was used to analyze the screened differential genes. Common differential genes of the OVX vs. SHAM and OVX + EX vs. OVX groups were analyzed using the Metascape website^2^ for gene ontology (GO) and signal pathway enrichment statistical analyses. The criterion for significance in screening was *P* < 0.01, with a corrected *p*-value < 0.05. Several key differential genes, including CCL2, were screened according to the FC, biological process, and pathway of the differential genes.

Total RNA was isolated from the PBMC samples using an Ultrapure RNA Kit (CWBiotech, Beijing, China), and the RNA was reversely transcribed into complementary DNA according to the instructions in the reverse transcription kit. Real-time quantitative polymerase chain reaction (qPCR) was performed in triplicate according to the instructions of the SYBR^TM^ Green PCR Master Mix Kit (CWBiotech, Beijing, China). The reaction system of the qPCR was 20 μl, and a random primer was used for the assay. The expression of each messenger RNA (mRNA) was expressed in multiples obtained using the 2 ^–ΔΔCT^ method and normalized to the housekeeping gene β-actin. Five genes were selected for mRNA verification. The primer sequences are presented in Table 1.

**TABLE 1:** The primer sequences for mRNA verification.

Gene Symbol	Upstream primer sequence	Downstream primer sequence
β-actin (Internal reference gene)	TGGCTCTAACAGT CCGCCTA	AGTGCGACGTGGAC ATCCG
LPL	AAACCCCAGCAAGGC ATACA	GTAGGGCATCTGAG AGCGAG
ITGB4	ACTGAGCACCTGGT GAATGG	CGCGTCAGAGAGTGG TAGTC
CCL2	GCCAACTCTCACTGAA GCCA	GACAGCACGTGGATG CTACA
TGFB3	CAGGATCTAGGCTGGA AATGGG	GTGCTGTGGGTTGTGT CTGAG
NOS3	GCTTGTTTCGCACAGG ATGG	TGAAAGGCAGGGACG TTGTT

### Statistical Analyses

Normally distributed variables are expressed as mean ± sd. A one-way analysis of variance was used to compare the differences between the three groups. When significant differences were identified, the Least Significant Difference method was used to perform multiple comparison tests between the groups. The correlation between the bone parameters and the differential gene expression was screened using the Pearson’s correlation analysis method. All *p*-values were two-tailed, and values less than 0.05 were considered to be statistically significant, with *P* < 0.01 representing a highly significant difference. Analyses were carried out using the SPSS software (version 23.0).

## Results

### Comparison of the Body Weight, Body Composition, and BMD of the Rats in Each Group

The body weight and fat content of the OVX group were significantly higher than the SHAM group, while the muscle content and BMD were significantly lower. The body weight and fat content of the OVX + EX group were lower than the OVX group, while the muscle content and BMD were significantly higher than the OVX group and significantly lower than SHAM group (see Table 2). These findings suggest that the ovariectomy led to an increase in body weight, body fat, and bone loss, while exercise helped to slow down the loss of muscle mass and bone density, partially mitigating the bone loss caused by the ovariectomy.

**TABLE 2:** Comparison of body weight, body composition and bone mineral density of rats in each group.

Indicators	SHAM	OVX	OVX + EX
Weight(g)	345.89 ± 34.05	456.75 ± 47.55**	425.43 ± 35.12**^ΔΔ^
Fat content of the whole body (%)	54.18 ± 6.86	65.37 ± 8.79**	58.51 ± 6.18^ΔΔ^
Muscle content of the whole body (%)	41.42 ± 7.04	29.40 ± 6.84**	38.36 ± 6.38^ΔΔ^
Upper limb fat%	3.70 ± 0.30	4.30 ± 0.58**	4.30 ± 0.41**
Lower limb fat%	9.80 ± 1.26	11.70 ± 1.90**	10.20 ± 1.86^ΔΔ^
Trunk fat mass%	29.80 ± 4.93	38.10 ± 4.56**	33.10 ± 4.88*^ΔΔ^
Upper limb muscle%	2.70 ± 0.49	2.20 ± 0.60**	2.70 ± 0.51^ΔΔ^
Lower limb muscle%	10.20 ± 1.19	8.00 ± 1.60**	9.20 ± 1.95^Δ^
Trunk muscle%	32.40 ± 4.79	25.30 ± 4.60**	29.40 ± 4.15*^ΔΔ^
Distal femur BMD(g/cm^2^)	0.280 ± 0.030	0.231 ± 0.022**	0.249 ± 0.020**^Δ^
Proximal tibia BMD(g/cm^2^)	0.224 ± 0.035	0.184 ± 0.031**	0.200 ± 0.021*
The 4th lumbar spine BMD(g/cm^2^)	0.238 ± 0.029	0.199 ± 0.029**	0.223 ± 0.023^ΔΔ^

Compared with SHAM, *means significant P < 0.05, **means significant P < 0.01; Compared with OVX, ^Δ^indicates significant P < 0.05, ^ΔΔ^indicates significant P < 0.01.

### Micro-CT Analysis and Three-Dimensional Reconstruction of the Distal Femur of the Rats in Each Group

**FIGURE 2:**
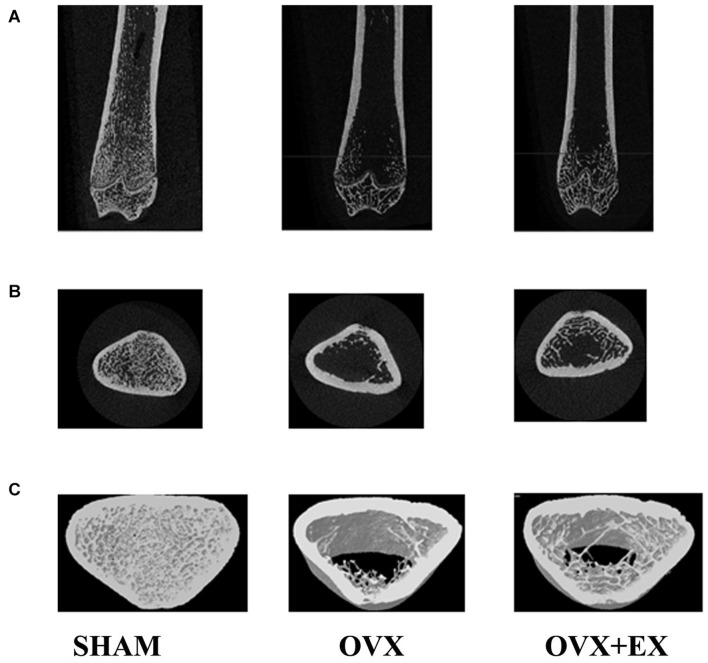
Two-dimensional structure of the longitudinal and transverse sections of the distal femur and the three-dimensional reconstruction of cancellous bone of the distal femur in the region of interest **(A)** the longitudinal section of the distal femur. **(B)** The transverse section of the distal femur. **(C)** 3Drec-onstruction of bone. Representative micro-CT pictures are shown.

When compared with the SHAM group, the mean total cross-sectional tissue area; closed porosity; BV ÷ TV; and the trabecular parameters including Tb.Th (pl), Tb.N (pl), Tb.N (rd), and Tb.Dm (rd) of the OVX rats were significantly lower. When compared with the OVX group, the Tb.Th (pl), Tb.Sp (rd), and Tb.Dm (rd) of the OVX + EX group were significantly higher, and the BV ÷ TV, Tb.N (pl), Tb.N (rd), and Tb.Pf were very significantly higher (see Table 3). The three-dimensional structure showed that the trabecular bones of the rats in the SHAM group were arranged evenly and tightly; the number of bone trabecula was dense, evenly distributed, and with good continuity; the trabecular bone space was narrow; and the shape was different. When compared with the SHAM group, the number of trabecular bones in the femur of the OVX group was significantly lower, the separation of trabecular bones was greater, and larger trabecular bone voids were formed locally. When compared with the OVX group, the number of trabecular bones in the OVX + EX group was greater, and the continuity was improved (see Figure 2). The results showed that the bone microstructure of ovariectomized rats showed osteoporosis, and moderate-intensity exercise increased the number and area of bone tissue per unit area and the number and thickness of bone trabecula, reduced the separation of the trabecular bones, and improved the beam changes from a rod shape to a plate shape and the bone microstructure of the femur.

**TABLE 3:** Comparison of distal femur parameters of rats in each group detected by micro-CT.

Indicators	SHAM	OVX	OVX + EX
Bone volume fraction (BV/TV)%	30.9890 ± 6.1263	1.9149 ± 0.9934**	8.7575 ± 0.5526**^ΔΔ^
Mean total cross-sectional tissue area μm^–2^	7.62E + 06 ± 1.20E + 06	6.73E + 06 ± 7.15E + 05	8.00E + 06 ± 7.79E + 05
Mean total cross-sectional tissue perimeter μm	1.46E + 04 ± 5.32E + 03	1.15E + 04 ± 8.97E + 02	1.26E + 04 ± 7.05 E + 02
Mean total cross-sectional bone area μm^–2^	3.18E + 06 ± 1.68E + 06	1.31E + 05 ± 7.80E + 04	7.00E + 05 ± 8.37E + 04
Mean total cross-sectional bone perimeter μm	6.10E + 04 ± 2.45E + 04	4.97E + 03 ± 2.81E + 03**	2.34E + 04 ± 3.73E + 03^Δ^
Mean number of objects per slice	56.50 ± 37.86	13.40 ± 6.46	40.80 ± 8.33
Closed porosity (percent)%	5.79 ± 2.45	0.03 ± 0.05**	0.20 ± 0.09^Δ^
Trabecular thickness [Tb.Th(pl)] μm	45.5903 ± 4.0018	33.9587 ± 2.6509**	40.5490 ± 2.7485*^Δ^
Trabecular separation [Tb.Sp(pl)] μm	103.9835 ± 20.5993	2089.3000 ± 1030.3506**	423.8033 ± 41.3892**^Δ^
Trabecular separation [Tb.Sp(rd)] μm	54.6435 ± 9.7143	396.3667 ± 107.5497**	162.0367 ± 13.5875**^Δ^
Trabecular number [Tb.N(pl)] μm^–1^	0.0067 ± 0.0007	0.0006 ± 0.0003**	0.0022 ± 0.0002**^ΔΔ^
Trabecular number [Tb.N(rd)] μm^–1^	0.0069 ± 0.00008	0.0022 ± 0.0005**	0.0041 ± 0.0004**^ΔΔ^
Diameter of trabecular bone [Tb.Dm(rd)] μm	91.1800 ± 8.0037	67.9170 ± 5.3022**	81.0983 ± 5.4966*^Δ^
Average of Trabecular Bone Pattern factor (Tb.Pf) μm^–1^	0.0026 ± 0.0022	0.0193 ± 0.0020**	0.0103 ± 0.0010**^ΔΔ^

Compared with SHAM, *means significant *P* < 0.05, **means significant *P* < 0.01; Compared with OVX, ^Δ^indicates significant *P* < 0.05, ^ΔΔ^indicates significant *P* < 0.01.

### Bioinformatics Analysis of the PBMC Transcriptome in Each Group

**FIGURE 3:**
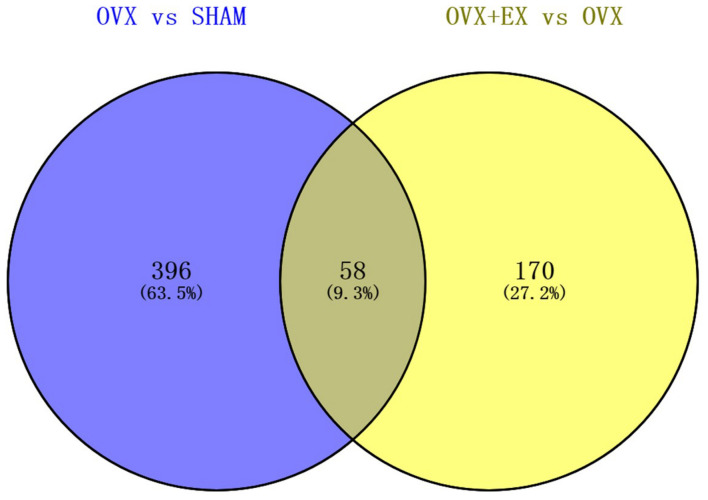
Venn diagram representing the overlap of DEGS in OVX vs. SHAM and OVX + EX vs. OVX.

**FIGURE 4:**
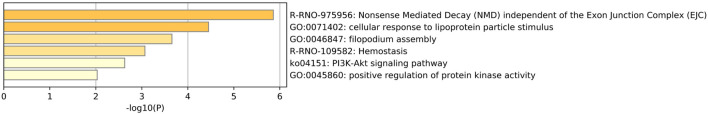
Go terms and KEGG pathways significantly enriched showing higher transcript levels in DEGS in OVX vs. SHAM and OVX + EX vs. OVX.

When compared with the SHAM group, there were 454 differentially expressed genes in the OVX group, including 114 upregulated genes and 340 downregulated genes. When compared with the OVX group, there were 228 differentially expressed genes in the OVX + EX group, including 128 upregulated genes and 100 downregulated genes. The Venn diagram shows that there were 58 common differential genes (see Figure 3). An enrichment analysis of the 58 differential genes showed that the main signaling pathway and biological processes enriched by the differential genes were the PI3K-Akt signaling pathway and the cellular response to lipoprotein particle stimulation (see Figure 4). According to the FC, biological processes, and pathways of the different genes, five key differential genes, including CCL2, were screened (see Table 4).

**TABLE 4:** Information of Key differential genes.

Gene symbols	Name of genes	Fold change (log_2_FC)	
		OVX vs. SHAM	OVX + EX vs. OVX
CCL2	chemokine (C-C motif) ligand 2	−3.673**	2.145^Δ^
Nos3	nitric oxide synthase 3	−3.034**	2.208^Δ^
Tgfb3	transforming growth factor beta 3	−1.278**	1.15^Δ^
ITGb4	integrin subunit beta 4	−1.217**	1.118^Δ^
LpL	lipoprotein lipase	−1.173*	1.099^ΔΔ^

Compared with SHAM, *means significant P < 0.05, **means significant P < 0.01; Compared with OVX, ^Δ^indicates significant P < 0.05, ^ΔΔ^indicates significant P < 0.01.

In terms of the mRNA expression levels between the groups, when compared with the SHAM group, CCL2, Nos3, and Tgfb3 were significantly lower in the OVX group, and Tgfb3 were lower in the OVX + EX group, and CCL2, Nos3, and LPL were significantly reduced; and when compared with the OVX group, CCL2, Nos3, Tgfb3, were higher in the OVX + EX group, and LPL significantly higher (see Table 5).

**TABLE 5:** The relative expression of Key differential genes mRNA.

	SHAM	OVX	OVX + EX
CCL2	1.78 ± 0.56	0.58 ± 0.20**	0.85 ± 0.11**^Δ^
Nos3	5.89 ± 2.49	1.02 ± 0.20**	1.86 ± 0.48**^Δ^
Tgfb3	2.44 ± 0.91	1.15 ± 0.16**	1.72 ± 0.23*^Δ^
ITGb4	1.21 ± 0.44	1.06 ± 0.09	1.32 ± 0.17
LPL	0.98 ± 0.36	0.60 ± 0.15	1.83 ± 0.34**^ΔΔ^

Compared with SHAM, *means significant P < 0.05, **means significant P < 0.01; Compared with OVX, ^Δ^indicates significant P < 0.05, ^ΔΔ^indicates significant P < 0.01.

### The Correlation Between Key Different Genes and the Distal Femur Parameters

A correlation analysis showed that the expression levels of the CCL2, TGFB3, and NOS3 genes were significantly positively correlated with multiple parameters of bone microstructure, and the results suggested that these three genes are important candidate biomarkers for the effect of exercise on osteoporosis (see Table 6).

**TABLE 6:** The correlation between key different genes and distal femur parameters.

	CCL2	NOS3	TGFB3	ITGB4	LPL
Bone volume fraction (BV/TV)%	0.966**	0.817**	0.845**	0.263	0.056
Mean total cross-sectional tissue area μm^–2^	–0.11	–0.19	–0.05	–0.36	0.158
Mean total cross-sectional tissue perimeter μm	0.638*	0.908**	0.668*	0.589	0.289
Mean total cross-sectional bone area μm^–2^	0.947**	0.949**	0.856**	0.425	0.119
Mean total cross-sectional bone perimeter μm	0.62	0.338	0.471	–0.2	–0.09
Mean number of objects per slice	0.188	0.01	0.071	–0.47	–0.03
Closed porosity (percent)%	0.955**	0.709*	0.832**	0.278	–0.09
Trabecular thickness [Tb.Th(pl)] μm	0.886**	0.708*	0.868**	0.533	0.425
Trabecular separation [Tb.Sp(pl)] μm	–0.57	–0.54	–0.6	–0.27	–0.5
Trabecular separation [Tb.Sp(rd)] μm	−0.712*	–0.65	−0.699*	–0.25	–0.41
Trabecular number [Tb.N(pl)] μm^–1^	0.933**	0.805**	0.798**	0.18	0.041
Trabecular number [Tb.N(rd)] μm^–1^	0.849**	0.765*	0.741*	0.128	0.138
Diameter of trabecular bone [Tb.Dm(rd)] μm	0.886**	0.708*	0.868**	0.533	0.425
Average of Trabecular Bone Pattern factor (Tb.Pf) μm^–1^	−0.864**	−0.749*	−0.830**	–0.33	–0.32

*means significant P < 0.05, **means significant P < 0.01.

## Discussion

### The Effect of Moderate-Intensity Treadmill Exercise on OVX Rats

Ovarian estrogen decreases significantly after menopause, and the lack of estrogen leads to obesity, decreased muscle mass, lipid metabolism disorders, decreased bone density, and other abnormal conditions. OVX rats are a typical experimental model for the study of postmenopausal osteoporosis due to estrogen deficiencies in women. It has been reported (Harding and Beck, 2017; Gomesa et al., 2018) that ovariectomy can lead to weight increase, obesity, dyslipidemia, and osteoporosis. Physical exercise has a positive significance for osteoporosis and can affect the quality of bone development in individuals of all ages, but BMD does not fully reflect bone quality. Mechanical load can also affect bone strength by affecting the geometric shape of the bone. Micro-CT refers to a medical CT with a spatial resolution of 100-1 μm, which can comprehensively measure the bone microstructure and evaluate bone quality. The structure of bone tissue mainly includes the thickness of the cortical bone and the density of its internal pores, as well as the shape, thickness, connectivity, and degree of anisotropy of the cancellous bone, i.e., the bone microstructure. The structural changes in the trabecular bone play an important role in bone strength (Dalle Carbonare and Giannini, 2004; Kleerekoper, 2006). After ovariectomy in rats, the pathological changes of osteoporosis mainly occurred in the cancellous bone (Rico et al., 1994). The decrease in the trabecular bone parameters, Tb.Th and Tb.N, and the increase in Tb.Sp are manifestations of osteoporosis. This study found that the bone microstructure of ovariectomized rats indicated osteoporosis, most notably the morphological structure of the bone trabecula was severely damaged. Sun et al. (2015) reported that a 60-day treadmill exercise routine resulted in an increase in BV ÷ TV, Tb.N, Tb.Th, BMD, and the mechanical strength of the femur in the OVX group. Kang et al. (2017) reported that 8 weeks of downhill training significantly reduced ovariectomy-induced bone loss, with uphill and downhill exercise being the most beneficial to bone formation. This study found that after ovariectomy, the rats that were given moderate-intensity treadmill exercise exhibited improved body composition, BMD, bone microstructure, and trabecular bone parameters, indicating that moderate-intensity treadmill exercise played a preventive role in the occurrence and development of osteoporosis. Previous literature (Ju et al., 2015; Cunha et al., 2018; Gomesa et al., 2018) has shown that swimming, strength training, resistance training, and other forms of exercise can improve body composition and bone mass in patients with PMOP and help prevent bone loss associated with PMOP.

Mesenchymal stromal cells (MSCs) are a type of stromal cell existing in most mature connective tissues, such as bone marrow, adipose tissue, and umbilical cord blood, and can be differentiated into osteoblasts, chondrocytes, and adipocytes under different physiological and pathological stimuli. Studies have shown that mechanical stimulation may promote osteogenesis by inhibiting the differentiation of MSCs into adipocytes. Recently, Liu et al. (2018) reported that moderate-intensity treadmill exercise enhanced the osteogenic potential, osteogenic differentiation, and maturation of MSCs in rats; upregulated the expression of mRNA in multiple osteogenic genes; and inhibited the adipogenesis of bone marrow MSCs. An analysis of the reasons for this suggested that there may be a biomechanical window for maintaining the best bone homeostasis, and the strain signal that is induced by exercise in the biomechanical window can lead to the proliferation and osteogenic differentiation of MSCs. In general, osteoporosis prevention training programs usually recommend moderate-intensity exercise (Beck et al., 2017). Li et al. (2014) reported that three-month wheel-running exercise inhibited the expression of interleukin (IL)-1, IL-6, and Cox-2 in the bone marrow of OVX rats and increased the bone mass of OVX rats by inhibiting trabecular bone resorption and increasing bone formation. Chen et al. (2011) found that the effect of moderate exercise on bone loss in OVX rats may be related to its inhibitory effect on peroxisome proliferator-activated receptor gamma (PPARγ), an adipogenic transcription factor, but not to runt-related transcription factor 2 (Runx2). However, Zahra et al. (Hemati Farsani et al., 2019) reported that there was no significant difference in the expression of microRNA (miR)-133a, miR-103a, miR-204, Runx2, and PPARγmRNA in the bone marrow of adult male Wistar rats after 8 weeks of strength and endurance training. Therefore, it is suggested that the influencing mechanism of exercise on PMOP is still unclear. The factors or pathways through which mechanical load stimulation affects the osteogenic and adipogenic differentiation of MSCs require further investigation.

### The Effect of Moderate-Intensity Treadmill Exercise on the PBMC Transcriptome of OVX Rats

It has been found that biomarkers in the PBMCs that can reflect bone changes are more advantageous in the sensitive prediction of bone remodeling dynamics and are easier to obtain and study than bone tissue in the human body. Mira et al. (Horváthová et al., 2017; Faienza et al., 2019) reported that age, BMI, BMD, waist circumference, and the tissue fat of postmenopausal women affected the lymphocyte immunophenotype. Many pro-inflammatory cytokines in obesity are also key participants in osteoclast formation and activation and participate in the changes of osteoporosis, indicating that obesity, cytokines, and bone turnover are related. In recent years, high-throughput technology has been used to explore the molecular mechanism of PMOP. Li et al. (2016) integrated several microarray gene expression datasets to screen for PMOP candidate genes in the PBMCs. Bhavna (Daswani and Ikram Khatkhatay, 2018) summarized the molecular list of BMD-related monocytes provided by omics technology, in which multiple cytokines and chemokines are involved. By analyzing the transcriptional profile of the peripheral white blood cells in young endurance athletes, Liu D. et al. (2017) supported the use of peripheral blood as an alternative tissue to evaluate the effects of exercise, and the PBMCs and several factors expressed in the PBMCs can be used to study the biological mechanism by which physical exercise affects people with PMOP, and PBMC gene expression may be a sensitive tool to characterize the early effects of exercise on bone regulation.

Contrepois et al. (2020) carried out the transcriptome analysis of the PBMCs after acute exercise. The enrichment analysis of the PBMC gene expression revealed many pathways that were negatively correlated with aerobic exercise. Calpain, integrin, and many inflammatory pathways were negatively correlated with VO_2_max, indicating that exercise changed the transcription spectrum of the PBMCs. This study showed that the PI3K-Akt signaling pathway and the cellular response to lipoprotein particle stimulation were involved in the process of changing the body composition and bone mass in OVX rats during moderate-intensity treadmill exercise. The PI3K-Akt signaling pathway is an important regulator of cell proliferation, metastasis, adhesion, and death. Under normal physiological conditions, the PI3K-Akt signaling pathway can selectively affect the physiological functions of osteoblasts and osteoclasts and plays an important role in maintaining the dynamic balance of bone tissue. *In vivo* and *in vitro* studies (Xi et al., 2015) have shown that the PI3K-Akt cell signaling pathway plays a role in the inhibition of osteoporosis by promoting the proliferation, differentiation, and bone formation of osteoblasts. Zhang et al. (2016) analyzed the protein expression profile of the PBMCs in 42 white women with PMOP and found that there were rich GO terms related to lipid binding. Genome enrichment analysis showed that the BMD of postmenopausal women may be affected by regulating the activity of proinflammatory cytokines. In this study, a transcriptome analysis of rat PBMCs showed that GO terms related to bone and lipid metabolism were abundant, and multiple genes related to bone or lipid metabolism, such as CCL2, appeared in the significant enrichment terms of GO and the signaling pathways. In this study, five genes related to bone or lipid metabolism, i.e., CCL2, Nos3, Tgfb3, ITGb4, and LpL, were screened. These may be important genes involved in the mechanism of moderate-intensity treadmill exercise in improving body composition and bone mass in OVX rats.

The PI3K-Akt pathway can be activated by growth factors and other extracellular signals to regulate many basic cellular processes, including cell growth, proliferation, differentiation, survival, and apoptosis. The members of the transforming growth factor beta (TGF-β) superfamily are effective regulators of homeostasis and bone repair *in vivo* and play a role in cell proliferation, osteogenic differentiation, osteoclast formation, and osteoblast/osteoclast balance (Jann et al., 2020). MacFarlane et al. (2017) proposed that TGF-β is a multifunctional cytokine closely related to bone metabolism and a potential marker to measure osteoporosis. Zhang et al. (2019) showed that TGF-β1 promoted the survival, osteogenic differentiation, and migration of osteoblasts through the PI3K/AKT/mTOR/S6 kinase 1 signaling pathway. In addition, TGF-β influences mTOR/S6K1 downstream of PI3K/AKT. This article showed that treadmill exercise significantly increased the level of TGF-βmRNA in OVX rats and was significantly positively correlated with bone microstructure parameters. The analysis showed that exercise promotes the expression of TGF-β, and TGF-β promotes osteoblasts through the PI3K/AKT pathway. Proliferation- and migration-related genes regulate bone formation and cell migration, thereby indicating an improvement in bone density and bone microstructure.

CCL2 promotes the recruitment of monocytes/macrophages into the intima and may be involved in regulating other signaling pathways related to atherosclerosis and metabolic disorders (Rull et al., 2010). CCL2 and its receptor, CCR2, are essential for the formation of osteoclasts and giant cells, and the deletion of these genes inhibits osteoclast formation (Khan et al., 2016). CCL2 in osteoblasts can effectively recruit osteoclast monocyte precursors and promote the nuclear factor kappa-B ligand-induced osteoclastogenesis receptor activator (Li X. et al., 2007) primarily to enhance fusion. CCL2 can accelerate the recruitment of osteoclast precursor cells and promote the differentiation and maturation of osteoclasts, thereby exerting a bone resorption function (Kyriakides et al., 2004; Siddiqui and Partridge, 2017). It has been reported that serum CCL2 levels can be used as a potential biomarker to reflect the severity of disease in patients with PMOP (Yang et al., 2016). Mechanical stress can also induce osteoblasts to express chemokines, leading to inflammation and bone remodeling. Studies have verified through wild-type mice that the induction of CXCL2 and CCL2 in osteoblasts may trigger bone remodeling under pressure (Maeda et al., 2015). Harmon et al. (2010) reported that CCL2 and other genes were upregulated after repeated exercise. This paper also found that moderate-intensity treadmill exercise increased the expression of CCL2 in OVX rats. Previous research (Lean et al., 2005) has found that oxidative stress caused by estrogen deficiency combined with decreased antioxidant enzyme activity, oxidation, and antioxidant imbalance played an important role in the pathological processes of osteoporosis. Nitric oxide synthase (NOS) is the main rate-limiting substance for the production of endogenous nitric oxide (NO). Osteoclasts, osteoclast precursors, osteoblasts, and osteocytes can all express NOS. Guo et al. (2013) reported that the plasma NO level in OVX osteoporosis rats and the expression of eNOS mRNA in the femur were decreased, which is consistent with the results of this study. The reason for this may be related to the elimination of oxidative stress induced by estrogen deficiency through moderate-intensity treadmill exercise. Gene expression levels showed that the expression levels of CCL2, Nos3, and Tgfb3 were significantly lower in the OVX group than in the SHAM group. Treadmill exercise significantly increased the levels of the CCL2, Nos3, and Tgfb3 genes. A correlation analysis revealed that the expression levels of CCL2, Nos3, and Tgfb3 were significantly positively correlated with multiple parameters of bone microstructure, suggesting that these three genes are involved in the occurrence of osteoporosis in OVX rats, and they are the sensitive genes for evaluating the effect of treadmill exercise in improving bone quality in ovariectomized rats.

It has been reported that the cell adhesion signaling pathway is an important mechanical transduction pathway involved in bone adaptation movement (Marie et al., 2014; Sontam et al., 2015). The effect of mechanical stress stimulation on osteoblast adhesion is primarily mediated by integrin (Takai et al., 2006), and the adhesion between cells and the extracellular matrix is accomplished by integrin. Zeng et al. (2016) analyzed the protein–RNA integration of monocytes from 33 white females, and the results revealed ITGA2B as a new gene related to osteoporosis. Lipoprotein lipase (LPL) is the key rate-limiting enzyme in triglyceride decomposition. Low estrogen levels hinder the activation of LPL, inhibit its fat decomposition function, and lead to the accumulation of fat. Ortmeyer et al. (2017) reported that aerobic exercise combined with diet can increase the LPL activity of the skeletal muscles and improve fatty acid metabolism in postmenopausal women. This study did not show that an ovariectomy reduced the expression of ITGb4 and LPL genes by RT-PCR, but it did show that moderate-intensity treadmill exercise increases the expression of LPL, which is not closely related to bone microstructure parameters. This result suggests that these genes may be not sensitive genes for indicating the improvement in the bone mass of OVX rats caused by moderate-intensity treadmill exercise.

In summary, moderate-intensity treadmill exercise improves body composition and bone mass and induces transcription changes of the PBMCs in the peripheral blood of OVX rats, which may be mediated by the activation of the PI3K/Akt signaling pathway. CCL2, Nos3, Tgfb3, and other bone/lipid-metabolism-related genes may be important candidate biomarkers for exercise in delaying the decline in bone mass of people with PMOP. Our study only found that multiple osteogenic and adipogenic genes were involved in the improvement of the body composition and bone mass of OVX rats induced by exercise at the transcriptional level. Other levels, such as protein, still need further study.

## Data Availability Statement

The original contributions presented in the study are publicly available. This data can be found here: National Center for Biotechnology Information (NCBI) BioProject database under accession number GSE184493.

## Ethics Statement

The animal study was reviewed and approved by Ethics Committee of Shandong Sport University (2018208).

## Author Contributions

LG conceived the idea and conceptualized the study. LG and YL collected the data and reviewed the manuscript. YL and Y-JY analyzed the data. LG and D-YZ drafted the manuscript. All authors read and approved the final draft.

## Conflict of Interest

The authors declare that the research was conducted in the absence of any commercial or financial relationships that could be construed as a potential conflict of interest.

## Publisher’s Note

All claims expressed in this article are solely those of the authors and do not necessarily represent those of their affiliated organizations, or those of the publisher, the editors and the reviewers. Any product that may be evaluated in this article, or claim that may be made by its manufacturer, is not guaranteed or endorsed by the publisher.

## Footnotes

1
http://bioinfogp.cnb.csic.es/tools/venny/index.html

2
http://metascape.org
